# Fabrication of inclined non-symmetrical periodic micro-structures using Direct Laser Interference Patterning

**DOI:** 10.1038/s41598-019-41902-x

**Published:** 2019-04-01

**Authors:** Sabri Alamri, Mikhael El-Khoury, Alfredo I. Aguilar-Morales, Sebastian Storm, Tim Kunze, Andrés F. Lasagni

**Affiliations:** 1 Fraunhofer-Institut für Werkstoff- und Strahltechnik IWS, Dresden, 01277 Germany; 2 Technische Universität Dresden, Institut für Fertigungstechnik, Dresden, 01062 Germany

## Abstract

The direct fabrication of microstructures, having a non-symmetrical morphology with controllable inclination, presents nowadays a challenging task. Natural examples of surfaces with inclined topographies have shown to provide anisotropic functionalities, which have attracted the interest of several researchers in the last years. This work presents a microfabrication technique for producing microstructures with a determined and controllable inclination angle using two-beam Direct Laser Interference Patterning. Polyimide foils are irradiated with a 4 ns UV (266 nm) laser source producing line-like structures with a period varying from 4.6 µm to 16.5 µm. The inclinations, retrieved by tilting the sample with respect to the optical axis of the setup, are changed from 0° to 75°, introducing a well controllable and defined inclination of the structure walls. The structuring parameters (laser fluence, number of laser pulses and interference period) as well as the inclination of the microstructures are correlated with the global tilting of the sample. As a result, a determined laser fluence and number of pulses are necessary to observe a remarkable non-symmetrical morphology of the structures. In addition, the presence of structural undercuts is reported, which opens the possibility for developing new direction-dependent properties on polymeric materials. As an example, preliminary results on light diffraction are presented, showing a similar behavior as blazed diffraction gratings.

## Introduction

Three-dimensional microstructures have been recently applied in microsystems such as micro-optical electronics, micro electro-mechanical as well as analytical systems^1^. Several applications of naturally-inspired structures exhibit an inclination or even an undercut, associated with remarkable wettability properties^2^, light extraction^3^ and specific friction and adhesion characteristics^4,5^. In particular, surfaces having a non-symmetrical topography, e.g. with a determined inclination angle can be used for instance to produce gecko-like synthetic adhesives, as recently demonstrated on SU-8 molds^5^. A well-known example from the animal-world where inclined structures play an important role is the snakes’ skin. For instance, squamate reptiles exhibit surface textures with asymmetric sub-micron and nano-scale features which are responsible for frictional effects, manifested in the reduction of adhesion^6^, increase of abrasion resistance^7^ as well as anisotropic frictional behavior^8^. Eventually, a well-controllable replication of the snake skin microstructures on technical surfaces therefore allows advanced surface functions for products and components.

Among the techniques able to create three-dimensional microstructures, different technologies are utilized such as two-photon polymerization^9^, micro stereo-lithography^10^, moving mask^11^ and inclined UV and X-ray lithography^12–14^. However, these methods exhibit some challenges: for instance the inclined exposure suffer from non-uniform patterning due to different distances from the energy source (e.g. UV light); the moving mask method presents problems with the precision of the moving stage, which affects the final surface morphology and micro-lens-based techniques often suffer from aberration effects due to the curvature of the micro-lenses^15^. Another possibility is the fabrication of inclined structures with mechanical techniques such as ultra-precision machining. Nevertheless, this method generally presents drawbacks such as the abrasion of cutting tools and a poor quality in surface finishing^16^.

An example of application of non-symmetrical structures are blazed diffraction gratings^17,18^. This particular type of gratings has the property of diffracting most of the light intensity in a defined diffraction order, which can be controlled by the inclination angle of the structures, their depth and periodicity. Such gratings are for instance used in spectroscopic applications^19^. A common production technique for blazed gratings is using a diamond tipped tool to cut parallel grooves into the coating on the substrate, or to impress an interference pattern on an inclined surface, coated with a photo-sensitive material^20^.

Although widely used in the microelectronics due to its excellent performances in terms of resolution, pattern homogeneity and reproducibility, UV lithography presents some technical drawbacks for specific applications. This originates from the fact that photocrosslinkable materials are needed, quartz masks with defined geometries must be previously fabricated and at least a subsequent developing process is required, thus increasing the processing costs. Moreover, UV lithography requires very clean processing conditions, cannot be easily applied to curved or three-dimensional surfaces and only a small set of materials is treatable, which reduces the availability of the technique to users dealing with diverse industrial applications and for which clean-room conditions are not applicable^1,21^.

Among the mask-free and single-step fabrication methods, laser micromachining achieved a dominant position in several industry-oriented fields. The conventional laser fabrication technique, known as Direct Laser Writing (DLW), is frequently used in the production of functional surfaces both on metals and polymers employing ultra-short laser pulses^22–27^. If coupled with scanning devices, DLW can achieve impressive performances in terms of scanning speed (e.g. 10–15 m/s) and tightly focused laser pulses enable micromachining with high precision and a spatial resolution down to a few tens of micrometers (10–30 µm)^28–30^. Nevertheless, like in other manufacturing techniques that employ a top-down structuring approach, the fabrication of inclined microstructures is a challenging task. However, projecting the laser beam at an oblique angle on the sample enables the fabrication of 2.5D features. This was shown by Wang *et al*., which introduced a turning mirror in the optical setup which resulted in surface textures with a defined inclination^31^. Although this approach enables an effective micro-sectioning with non-vertical sidewalls profiles and high penetration depths (up to 380 µm), the minimal achievable lateral feature resolution is strongly limited by the focal length (75 mm) and thus restricted to some hundreds of micrometers^31^.

An innovative technique that allows the fabrication of periodic microstructures on different materials is Direct Laser Interference Patterning (DLIP). DLIP relies on the overlap of multiple coherent laser beams in order to generate interference patterns within the laser beam profile, with resolutions in the micro- and sub-micrometer scale. The direct application of the generated interference pattern on materials results in a well-defined surface textures. It can be demonstrated that the number of interfering laser beams, their geometrical arrangement, individual angle of incidence, phase and polarization influence the shape of the interference pattern^32^. The lateral dimension of the periodic pattern (spatial period Λ) can be controlled by the intercepting angle between the individual sub-beams, as described in Eq. 1 for a two-beam DLIP setup by

1 $$\Lambda =\frac{\lambda }{2\,\sin (\beta )}$$

where *λ* and *β* denotes the laser wavelength and half-angle between interfering beams, respectively. The DLIP technology is capable of treating a wide number of materials, ranging from metals to polymers and coatings^33–39^ with processing speeds up to 0.9 m²/min^40^. Moreover, this method has been employed in many application fields, such as to reduce friction on metals, to improve the adhesion of bone cells for dental implants, to fabricate nanoparticles for photocatalysis enhancement, for growing ZnO nanowires for sensing applications, to change the wettability on metals and polymers as well as for improving the conductivity in spot welding^37,41–45^.

In this work, the fabrication process of inclined DLIP microstructures is introduced, employing a conventional setup for interference structuring and a manual tilting stage for controlling the sample inclination. The aim of the work is to show the fabrication feasibility of periodical microstructures with a controllable inclination, depth and spatial period. As example of non-symmetrical properties, preliminary results on light diffraction are presented. The treated surfaces are characterized using confocal and scanning electron microscopy.

## Results and Discussion

### Fabrication of inclined structures using DLIP

A two-beam DLIP setup has been employed for the structuring of Polyimide (PI) foils which results in the fabrication of line-like surface structures. The employed laser source emits UV radiation (266 nm), which ensures a high absorption by the PI foils. Furthermore, due to the short wavelength used, the produced radiation  have a high photon energy, leading to the photochemical ablation of the polymer with a negligible contribution of photothermal processes^46,47^.

The processing parameters were fixed to a laser fluence of 1.32 J/cm² and 20 laser pulses per area, while the setup was adjusted to have an interference angle 2*β* of 3.28°, which results in a spatial period of 4.6 µm. As Eq. 1 shows, larger interference angles produce smaller spatial periods. However, a compromise between the interference periods and the inclination angles must be found. In fact, considering for example an interference angle 2*β* of 90°, the maximal possible inclination angle would be 45°. In addition, for large interference angles larger differences in the projected areas of the sub-beams occur, meaning that additional optical elements (such cylindrical lenses) are needed for compensating the variation in the areas (and thus fluences).

The choice of the initial structuring conditions employed in this work (laser wavelength, spatial period and laser fluence) derives from the necessity to obtain well-defined and regular ablation profiles. In fact, at a wavelength of 266 nm (corresponding to the laser radiation) this material presents a high absorption coefficient and the main interaction mechanism with the laser beam results in a photochemical ablation process without thermal heating, leading to high-quality patterns, as already reported by Lasagni *et al*.^36^. Moreover, the used laser fluence ensured a high ablation depth within a single laser pulse (~0.5 µm). The reference values have been taken from previous works^34,48^. The interference period was set to 4.6 µm, in order to avoid undesired effects, such unselective ablation (resulting from the expansion of the plasma plume) as previously investigated on polycarbonate and PET^49,50^, or lowering of the structure quality employing sub-micrometer patterns^51–53^.

**Figure 1:**
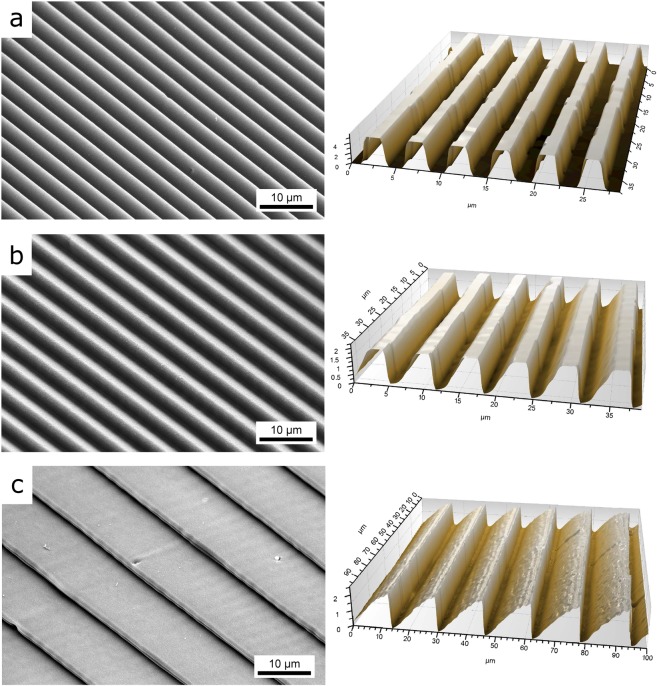
SEM micrographs (left) and confocal topography (right) of the structured polyimide foils using a spatial period of 4.6 µm, a laser fluence of 1.32 J/cm², 20 pulses per laser spot and different sample inclinations: (**a**) 0°, (**b**) 45° and (**c**) 75°.

The effect of interference pattern inclination on the material surface was investigated for inclination angles of 0, 30, 45, 60 and 75°. Examples of the produced structures with different inclination angles are shown in the Scanning Electron Microscope (SEM) images and confocal profiles depicted in Fig. 1. As it can be easily recognized, a tilt in the shape of the line-like structures is significantly visible for inclination angles *φ* greater than 30°. The SEM images also indicate the presence of an undercut of the inclined walls, which cannot be evaluated from confocal microscopies because of the top-view approach of the optical measurement itself.

**Figure 2:**
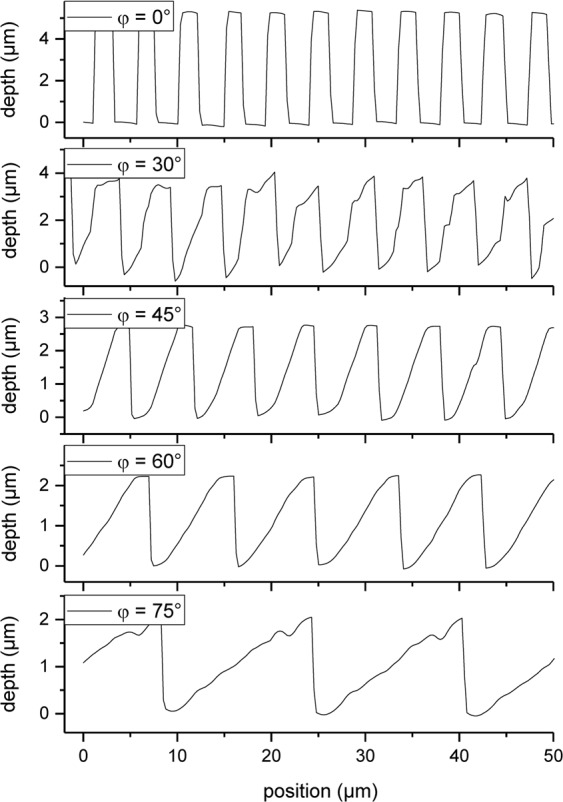
Change of the topography shape as a function of the inclination angle for fixed DLIP processing conditions (spatial period: 4.6 µm, fluence: 1.32 J/cm² and 20 pulses per laser spot).

From the confocal measurements presented in Fig. 1, a detailed analysis of the topography shape evolution in dependence on the inclination angles was performed, as shown in Fig. 2. Beside the tilt of the surface textures, the inclination of the interference pattern with respect to the sample surface also influences the periodicity and depth: for increasing inclination angles the spatial period increases and the structure depth decreases.

Both the change in spatial period and structure depth can be explained by considering the projection of the interference pattern on an inclined plane. The relation between the spatial period Λ and the inclination angle φ can be described by Eq. 2, where Λ_0_ is the spatial period for zero inclination (φ = 0°).

2 $${\rm{\Lambda }}({\rm{\phi }})=\frac{{{\rm{\Lambda }}}_{0}}{\cos \,{\rm{\phi }}\,}$$

**Figure 3:**
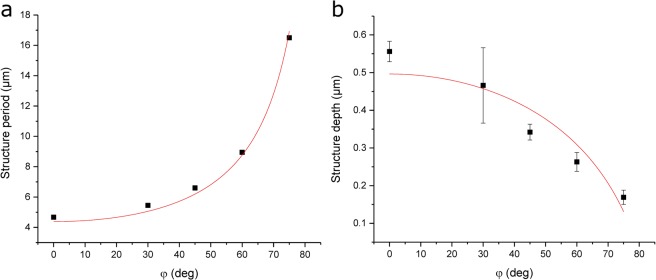
(**a**) Spatial period and (**b**) structure depth as a function of the sample’s inclination. The solid lines in (**a**) and (**b**) are fits corresponding to Eqs 2 and 5, respectively. The used laser fluence was 1.32 J/cm² with 1 pulse per laser spot.

Figure 3a shows the relationship between the measured periodicities and the tilting angle, which exemplifies that larger inclination angles φ result in a larger spatial period. It can be seen, that the experimental results are in very good agreement with the predicted periodicities using Eq. 2. The same projection principle can be also applied in order to calculate the effective interference area A as a function of the inclination angle, as given by Eq. 3:

3 $$A(\phi )=\frac{{A}_{0}}{\cos \,\phi }$$

where A_0_ is the area of the interference region for zero inclination (φ = 0°). The change in the interference area impacts also the effective laser fluence *F* irradiating the material’s surface, as described by Eq. 4 as a function of the inclination angle:

4 $$F(\phi )=\frac{E}{A(\phi )}=\frac{E}{{A}_{0}}\,\cos \,\phi ={F}_{0}\,\cos \,\phi $$

where E is the pulse energy and F_0_ is the laser fluence for zero inclination (φ = 0°).

It is known that for polymers, which are ablated following a photo-chemical ablation process, the structure depth is directly related to the wavelength-specific absorption depth and the employed laser fluence through the Lambert-Beer law^54^. Thus, the change in the laser fluence introduced by the different inclination angles results in changes in the structure depth *d*. Consequently, by considering Eq. 4, the Lambert-Beer law can be rewritten as:

5 $$d(\phi )=\frac{1}{\alpha }\,\mathrm{ln}\,\frac{F(\phi )}{{F}_{th}}=\frac{1}{\alpha }\,\mathrm{ln}(\frac{{F}_{0}}{{F}_{th}}\,\cos \,\phi )={d}_{0}+\frac{1}{\alpha }\,\mathrm{ln}(\cos \,\phi )$$

where α is the absorption coefficient at the used laser wavelength and d_0_ is the depth for a defined laser fluence F_0_ at φ = 0°.

The model outlined in Eq. 5 was verified by measuring the experimental structure depths as a function of the inclination angle for single DLIP pulses, as shown in Fig. 3b. Note that the theoretical prediction and the experimental results are in fair agreement. By fitting the experimental results to Eq. 5, an absorption coefficient of $3.7\pm 0.77\cdot {10}^{4}\,c{m}^{-1}$ can be calculated which is comparable to other reported data in the literature^55–57^.

Using a rectangular mask and a constant intensity distribution to irradiate the material, very well defined areas could be processed, especially for low inclination angles. However, for large angles (>60°) the outer regions of the treated areas present some irregularities (see Fig. S1 in the supplementary information section). For an inclination angle of 75°, these defects were observed in ~100 µm (for each side) of the total treated length (1.95 mm), representing around 10% of the total area. This effect can be attributed to the different size of the two interfering beams, while being projected on the same inclined surface. In particular, since the beams illuminate the surface with an interference angle *β*, the projection of the single beams is equivalent to a projection with angles φ + β and φ − β, where φ is the sample’s inclination angle. This produces a difference in the projected area of the beams and thus the overlap area is smaller than the size of single beams, lowering the interference contrast in the outer regions of the DLIP-treated areas.

### Control of the structure inclination

In order to evaluate the most adequate processing conditions to obtain deep and well-defined non-symmetrical patterns with a sawtooth morphology, further DLIP experiments were performed with variations in the number of laser pulses. Microstructures with high depths are commonly achieved either by overlapping sequential pulses^58,59^ or by means of multiple irradiations in the same area^60^. While the first approach is preferable when using small laser beam sizes and covering large areas in a homogeneous way^61^, the latter is used in case of large beam sizes and with laser-systems employing low repetition rates, like the system employed in this work.

**Figure 4:**
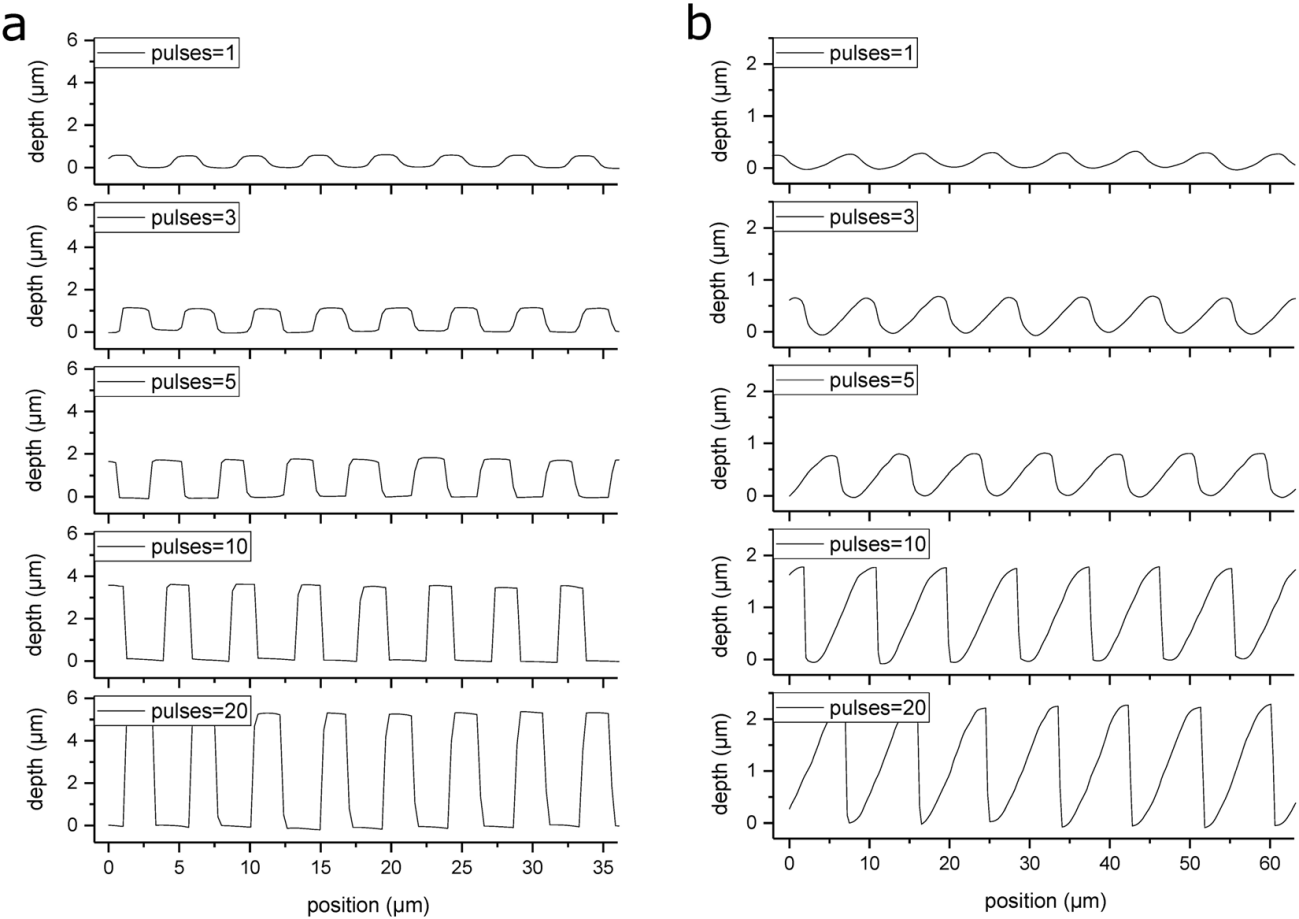
Variation of the structure morphology as a function of the number of laser pulses (1–20) for (**a**) normal irradiation and (**b**) 60° inclined irradiation, using a laser fluence of 1.32 J/cm².

Figure 4a shows the topography of irradiated PI samples with varying number of laser pulses (between 1 and 20), a constant laser fluence of 1.32 J/cm² and for perpendicular irradiation conditions (φ = 0°). As it can be seen, increasing the number of laser pulses results in an increase of the structure depth while the shape of the periodic structures evolves from sine-like to square-like with aspect ratios higher than 1. Note that the aspect ratio is defined as the quotient between the structure depth and the spatial period. For example, by applying 20 laser pulses, a structure depth of 5.3 µm was obtained for a spatial period of 4.6 µm, resulting in an aspect ratio of 1.15 (compare the profiles in Fig. 4a).

Figure 4b show the structure shapes of the DLIP patterns under an inclination angle of 60° for different number of pulses. It can be seen that the pattern morphology changes from sine-like to sawtooth-like. Furthermore, the sawtooth morphology character is more defined when three or more laser pulses are used to irradiate the sample. However, for a low number of pulses (in this case below three pulses) the inclination of the structure is not clearly recognizable. This behavior is attributed to the fact that for low laser fluences, the amount of ablated material is limited to a few hundreds of nanometers under the polymer surface, which makes not possible to follow the incoming inclined sine-like intensity distribution during the ablation process.

**Figure 5:**
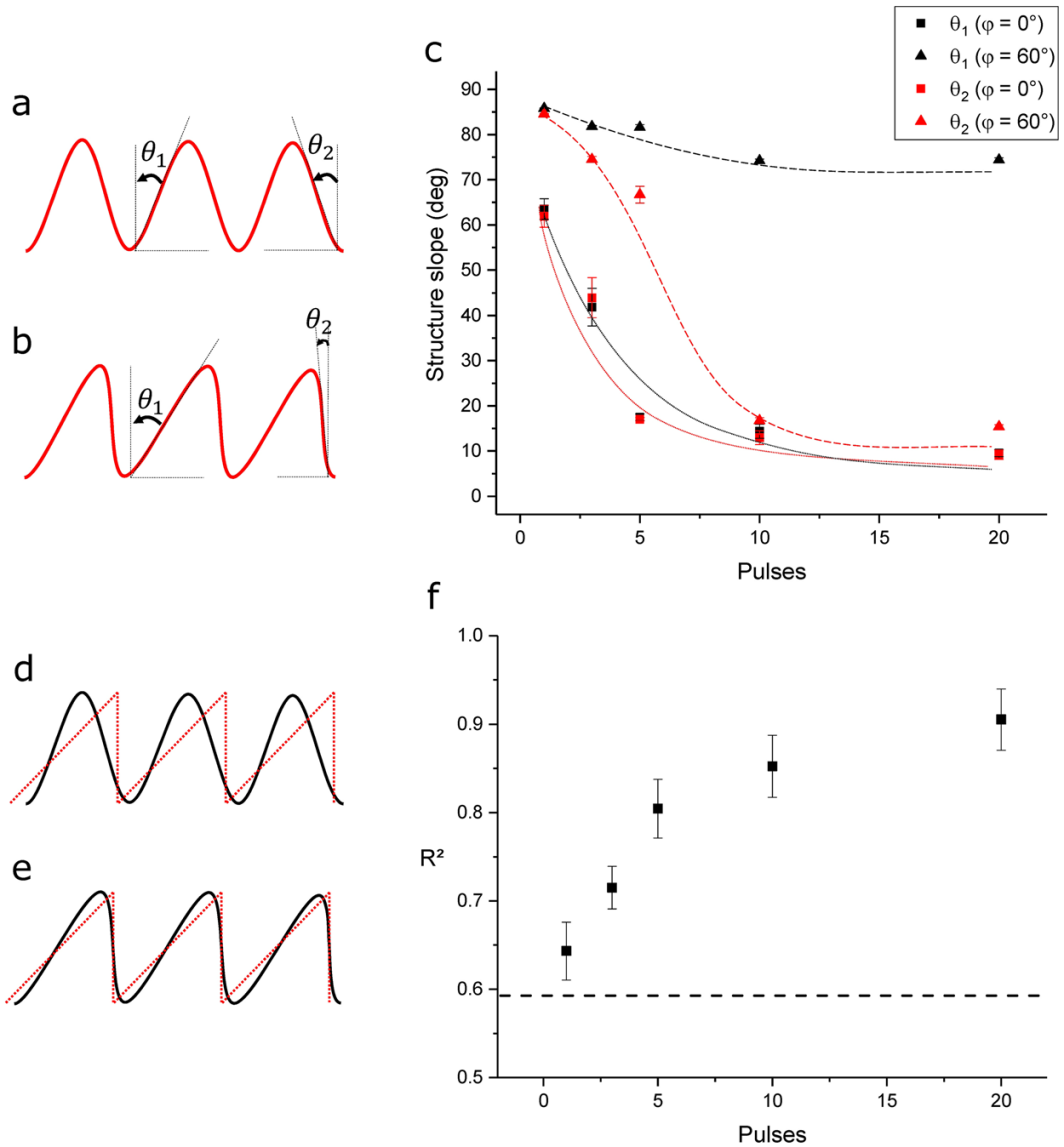
Schematic representation of the structure angle *θ*_1_ and undercut angle *θ*_2_ for a sine-like wave (**a**) and a saw tooth-like structure (**b**); evolution of the structure *θ*_1_ and undercut angles *θ*_2_ as function of the number of laser pulses (**c**) (dotted and solid lines serve as guides to the eye only); schematic representation of the fit of a sine-like wave (**d**) and a saw tooth-like wave (**e**) with a saw tooth wave function; variation of the R² coefficient as a function of the laser pulses for the fit of the structure profiles obtained with an inclination of 60° with a saw tooth wave function (**f**).

For a better description of the inclination of the produced topographies, two different angles were defined for the produced profiles, which are related to the structure shape. These angles consider the inclination angle of the structures (*θ*_1_) and the undercut angle (*θ*_2_). A scheme describing these two angles is shown in Fig. 5a and b for the case of a symmetrical (sinusoidal) and an asymmetrical (sawtooth-like) profile geometry, respectively. In the case of a sinusoidal curve, *θ*_1_ and *θ*_2_ have the same value of 67.3°, while *θ*_1_ is higher than *θ*_2_ for right-oriented saw tooth-like structures (compare Fig. 5b). The structure angles *θ*_1_ and *θ*_2_ have been retrieved directly from the measured confocal profiles. Figure 5c summarizes *θ*_1_ and *θ*_2_ for the case of normal incidence (squares) and an inclination angle of 60° (triangles) as a function of the number of pulses.

As it can be seen from Fig. 5c, the structures exhibit similar slopes in both directions in the case of the single pulse structuring and its values approach the theoretical ones for a sinusoidal profile (67.3°). Then, the two angles evolve in different directions when the number of pulses is increased, depending on the imposed sample inclination. For the symmetrical structures, both structure angles decrease with the number of pulses indicating that the structure shape changes towards a square-wave-like profile (similarly to what has been presented in Fig. 4a). On the other hand, for the structures fabricated with an inclination angle of 60°, the *θ*_1_ angle slightly decreases while the *θ*_2_ angle reaches very low values (~10°) when several laser pulse are used to irradiate the PI foil, which indicates a shape change from sinusoidal to saw-tooth like. It has to be mentioned that due to the characteristics of the optical method (confocal microscopy) used for measuring these angles, the undercut angle *θ*_2_ reaches a saturation towards 10° for increasing laser pulses, since undercuts cannot be evaluated (negative values).

As it can be seen from the profiles in Fig. 4b, the shape of the microstructures is affected with increasing number of laser pulses, changing from a sine to a saw tooth-like morphology. However, due to the ablation process, at the base positions of the saw tooth geometry, the profiles are smoother compared to a perfectly triangular wave. In order to quantify any deviation from a perfect saw tooth wave (see Fig. 5d,e), the profiles showed in Fig. 4a were fitted with a saw tooth wave function and their respective coefficient of determination (R²) was plotted as function of the number of laser pulses in Fig. 5e (R^2^ = 1 denotes a perfect saw tooth wave). It can be noticed that with increasing number of laser pulses the R² values uniformly rises up to 0.9 (for 20 pulses), denoting a strong saw tooth-like morphology. In addition, the fit of a perfect sine function (Fig. 5d) yields a R² of approximately 0.59 (dashed line in the graph), which is very similar to the R² value calculated for the surfaces treated with only 1 laser pulse (R² = 0.64).

**Figure 6:**
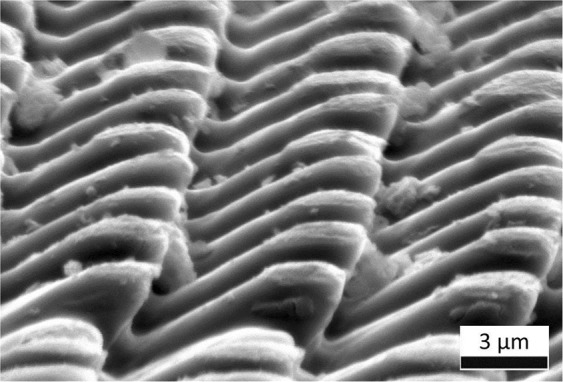
Inclined pillar-like structures obtained by a double irradiation setup employing a laser fluence of 1.32 J/cm², 20 pulses per area and an inclination of 45° for both irradiation steps.

For a better visualization of the undercut structures (with negative *θ*_2_ angles), a PI sample processed at a laser fluence of 1.32 J/cm² with 20 pulses and an inclination of 45° was re-irradiated using the same DLIP setup after sample rotation of 90°. In this way, an inclined pillar-like pattern was produced which permits observing the undercuts (*θ*_2_ < 0) of the micro-pillars, as shown in Fig. 6.

**Figure 7:**
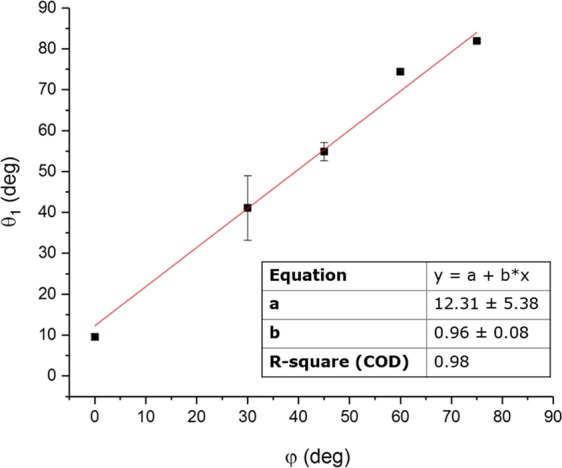
Variation of the structure inclination angle *θ*_1_ as a function of the sample inclination *φ*.

Due to the remarkable inclination of the obtained patterns when using 20 laser pulses, the analysis of the structure angles has been performed also other inclination angles. In particular, the evolution of the structure inclination (angle *θ*_1_) as a function of the sample inclination (*φ*) was studied. The results shown in Fig. 7 indicate a linear correlation between these two angles. Furthermore, it can be seen that the measured structure inclination angle (*θ*_1_) is slightly higher than the sample inclination (*φ*). In fact, the results indicated that for the normal irradiation conditions (inclination angle *φ* = 0°), a structural angle *θ*_1_ of 10° was observed. This behavior is attributed to the fact that for perpendicular irradiation, although the structures have a high depth and the structure walls are steep, the irradiation through a sine-like intensity distribution creates smoother profiles than the ones achievable with an ideal square-wave profile.

In order to show an example of asymmetrical macroscopic properties, the optical diffraction properties of the treated surfaces were evaluated using linearly polarized and monochromatic laser light (λ = 632 nm).

### Measurements of light diffraction of the textured PI foils

**Figure 8:**
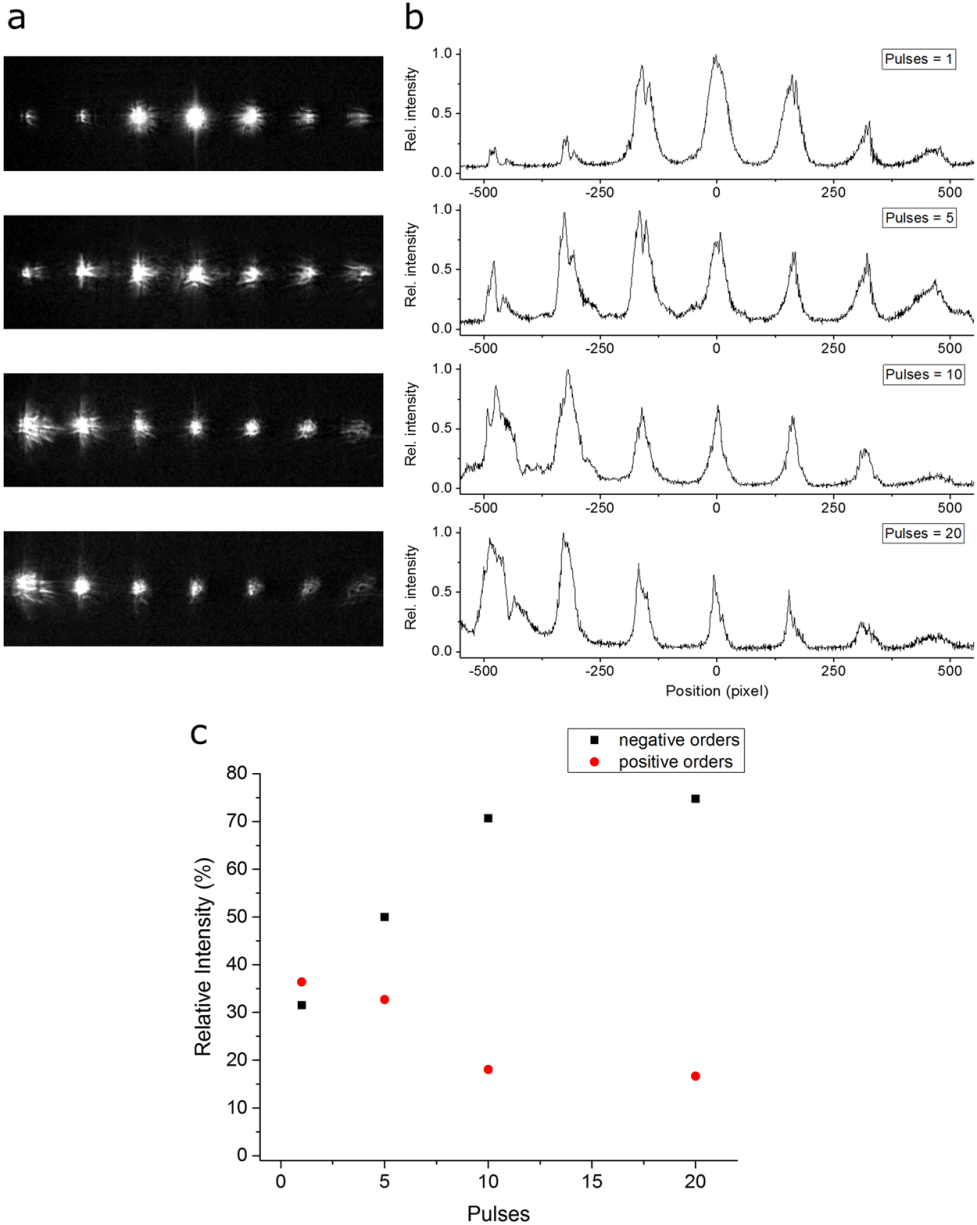
(**a**) CCD-Images of the diffraction and (**b**) diffractograms of the inclined structures produced with an inclination of 60° for different laser pulses, and (**c**) relative diffraction intensity of the negative (*I*^−^) and positive (*I*^+^) orders as a function of the laser pulses.

After their fabrication, the inclined microstructures were characterized in terms of their optical diffraction properties, using monochromatic laser light (λ = 632 nm). The diffractograms reported in Fig. 8b, show the evolution of the diffraction pattern as a function of the used number of laser pulses and thus depending on the inclination angle of the structures. Note that the patterns correspond to the same structures presented in Fig. 4b (i.e. fluence of 1.32 J/cm², 20 pulses per area and *φ* = 60°). In Fig. 8a,b it can be seen that an increase of the number of pulses results in a shift of the intensity of the overall diffracted light towards the negative diffraction orders (left direction). This effect can be clearly correlated with the decrease of the slope of the inclined structures (see Fig. 5). In order to quantify the magnitude of the shift, the diffractograms were fitted with Gaussian peaks and the intensity of each peak was used for calculating the amount of intensity diffracted into the negative and positive orders, as described in Eqs 6 and 7:

6 $${I}^{-}=\frac{{\sum }_{i}{I}_{i}^{-}}{{\sum }_{i}{I}_{i}}$$

7 $${I}^{+}=\frac{{\sum }_{i}{I}_{i}^{+}}{{\sum }_{i}{I}_{i}}$$

where, ${I}_{i}^{-}$ and ${I}_{i}^{+}$ are intensities of the negative and positive diffraction orders, respectively, while ${\sum }_{i}{I}_{i}$ is the sum of the intensity of all diffraction orders.

Consequently, *I*^−^ and *I*^+^ represent the amount of diffracted light directed in the negative and positive orders, respectively. The variation of the relative intensities *I*^−^ and *I*^+^ has been plotted as a function of the number of pulses employed for the inclined structuring, as depicted in Fig. 8c. Similarly to the behavior of blazed gratings, part of the diffracted light shifts towards the negative orders, which corresponds to the same direction in which the structures are inclined. In particular, it can be seen that for a high number of pulses, where the structure inclination is more defined, more than 70% of the diffracted light is directed in the negative orders, while ~15% of the light generates positive diffraction orders.

## Summary and Conclusions

In this work, asymmetrical line-like patterns with a sawtooth morphology were produced on polyimide foils by tilting the samples under irradiation with a two-beam interference setup. Compared to the orthogonal irradiation condition, an increase of the inclination angle resulted in larger periodicities. Since the size of the irradiated area also increases with the inclination angle, lower laser fluences are obtained at the material’s surface and thus a decrease in the structure depth is produced.

In order to quantify the inclination of the structures, two angles were defined, namely the structure and undercut angle. A direct correlation between the number of laser pulses and the two angles has been found, making possible to correlate the structuring process with the fabricated microstructures. On the other hand, a linear correlation between the sample inclination and the structure angle was observed, demonstrating the direct relation between structuring conditions and the shape of the structures.

As a direct result of the asymmetrical morphology of the structures, the optical properties of the structured samples were affected. In particular, non-symmetrical diffraction patterns were collected, with more than 70% of the diffracted light in the negative orders. In future experiments, the relationship between the surface morphology with additional surface functions such wettability and friction will be investigated in detail.

## Materials and Methods

**Figure 9:**
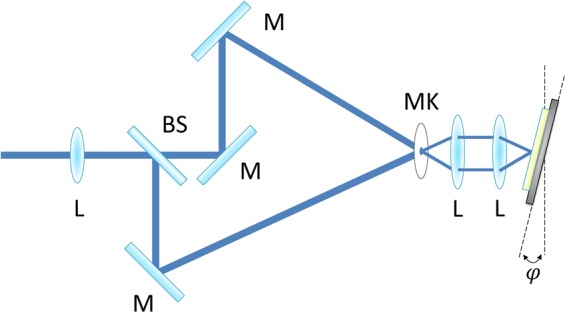
Top-view of the experimental setup used to irradiate the polymer samples with a two-beam interference pattern under an inclination angle **φ**.

For the DLIP structuring experiments, commercial polyimide (PI) foils (thickness of 125 µm, Goodfellow GmbH, Germany) were used. This material has been chosen due to the already reported photochemical ablation characteristic at UV wavelengths, permitting to obtain periodic structures with a remarkable quality^53^. For all the conducted experiments, a two-beam interference setup was utilized to produce line-like surface patterns, using a UV (266 nm) multimode Q-switched Nd:YAG solid state laser (Spectra Physics, Quanta Ray) with a pulse duration of 4 ns and a repetition rate of 10 Hz. In the experimental setup shown in Fig. 9, the primary laser beam was divided into two coherent beams using a 50% reflective beam splitter (BS). The mirrors (M) were positioned to ensure that the laser beams overlap on a ceramic mask (MK), which crops a homogenous squared area of 0.5 mm × 0.5 mm. The beams continue the path diverging from the mask and are parallelized through a converging 100 mm lens (L) and re-overlapped by another converging lens with a 100 mm focal distance. This configuration permitted to form the image of the mask over the sample surface. No control of the feedback of the interference phase has been applied, since the interference is generated by the intersection of the wave-fronts and the angle generating this is not varying during the irradiation processes. The distance between the mirrors as well as the position of the lenses, has been adjusted in order to achieve a spatial period of 4.6 µm. To cover larger areas, the substrate was translated by means of three motorized linear stages (Aerotech PRO165LM) in X, Y and Z directions. The optical configuration was placed on an optical table equipped with vibration isolators (NewPort S-2000)  and  laminar flow damping, which guarantees horizontal and vertical isolations of 85% and 94% at 5 Hz, respectively. During the irradiation process, the sample was translated at constant speed of 10 mm/s. Taking into consideration a pulse duration of 4 ns, this corresponds to a distance of 0.04 nm, and thus the movement of the sample during the laser treatment is negligible. In addition, for multiple laser pulses the sample is not moved until the completion of the firing events. Although vibrations of the optical components, fluctuations in the air refraction index and thermal drifts can occur, and thus leading to a shift of the interface phase (i.e. a lateral shift of the interference pattern)^62^, any additional system for locking the interference phase has been used in the experimental setup. The reason is mainly due to the very short duration of the used laser pulses (4 ns), which means that the interaction time during exposure is much shorter than any possible vibration period taking place on the employed setup.

For the experiments, the tilting of the sample was imposed by inclining the sample plane by means of a manual tilting stage (Thorlabs AP180/M). Although this requires that the axis movements must be coordinated in order to keep the interference area on the sample surface, this procedure allows using conventional DLIP setups, without any optical modification. The morphology of structured samples was characterized using confocal microscopy (Sensofar S Neox) employing a 50x magnification objective with a nominal lateral and vertical resolutions of 340 nm and 4 nm, respectively. Topographical inspections have been carried out also by means of Scanning Electron Microscopy (JEOL JSM 6610LV), coating previously the PI samples with a 30 nm thick gold layer. The measurements of the angles associated to the produced structures have been performed from the acquired confocal profiles. Using the software LeicaMap, six single profiles were analyzed, retrieving the slopes for both angles as well as their standard deviations, employed as error bars on the plots. The angles are obtained by fitting the profile of the ridges (cross-sections of the line-like structures) on both sides using linear equations and calculating their slopes. The structure profiles were compared to a saw tooth wave function by fitting their shape, keeping the spatial period constant as a fit parameter and retrieving the R² coefficient. This procedure was repeated on six different profiles and the standard deviation of the obtained R² values have been taken in consideration and plotted as error bars.

The diffraction properties of the laser treated polymers were measured by irradiating the samples with a red (He-Ne, 632 nm, linearly polarized) laser source, with a beam diameter of ~1 mm. The red laser beam hit the patterned areas orthogonally to the sample surface and its polarization was parallel to the DLIP line-like structures. The intensity distribution of the diffraction spectra was recorded in transmission on a CCD camera, employing an imaging system consisting on a focusing lens (with 50 mm focal length) and an objective with a magnification factor of 20X.

## Supplementary information

Figure S1
