# Supplementary material for: Fabrication of inclined non-symmetrical periodic micro-structures using Direct Laser Interference Patterning
Source: Sci Rep. 2019 Apr 1;9:5455. doi: 10.1038/s41598-019-41902-x (PMC6443938; doi:10.1038/s41598-019-41902-x)
Supplement: Supplementary file 1 — Figure S1 [file 41598_2019_41902_MOESM1_ESM.docx]

**Supplementary Information for the manuscript:**

**Fabrication of inclined non-symmetrical periodic micro-structures using Direct Laser Interference Patterning**

**Sabri Alamri**1,***, Mikhael El-Khoury**1**, Alfredo I. Aguilar-Morales**1**, Sebastian Storm**1,2**, Tim Kunze**1**, Andrés F. Lasagni**1,2

^1^ Fraunhofer-Institut für Werkstoff- und Strahltechnik IWS, Dresden, 01277, Germany

^2^ Technische Universität Dresden, Institut für Fertigungstechnik, Dresden, 01062, Germany

*sabri.alamri@iws.fraunhofer.de


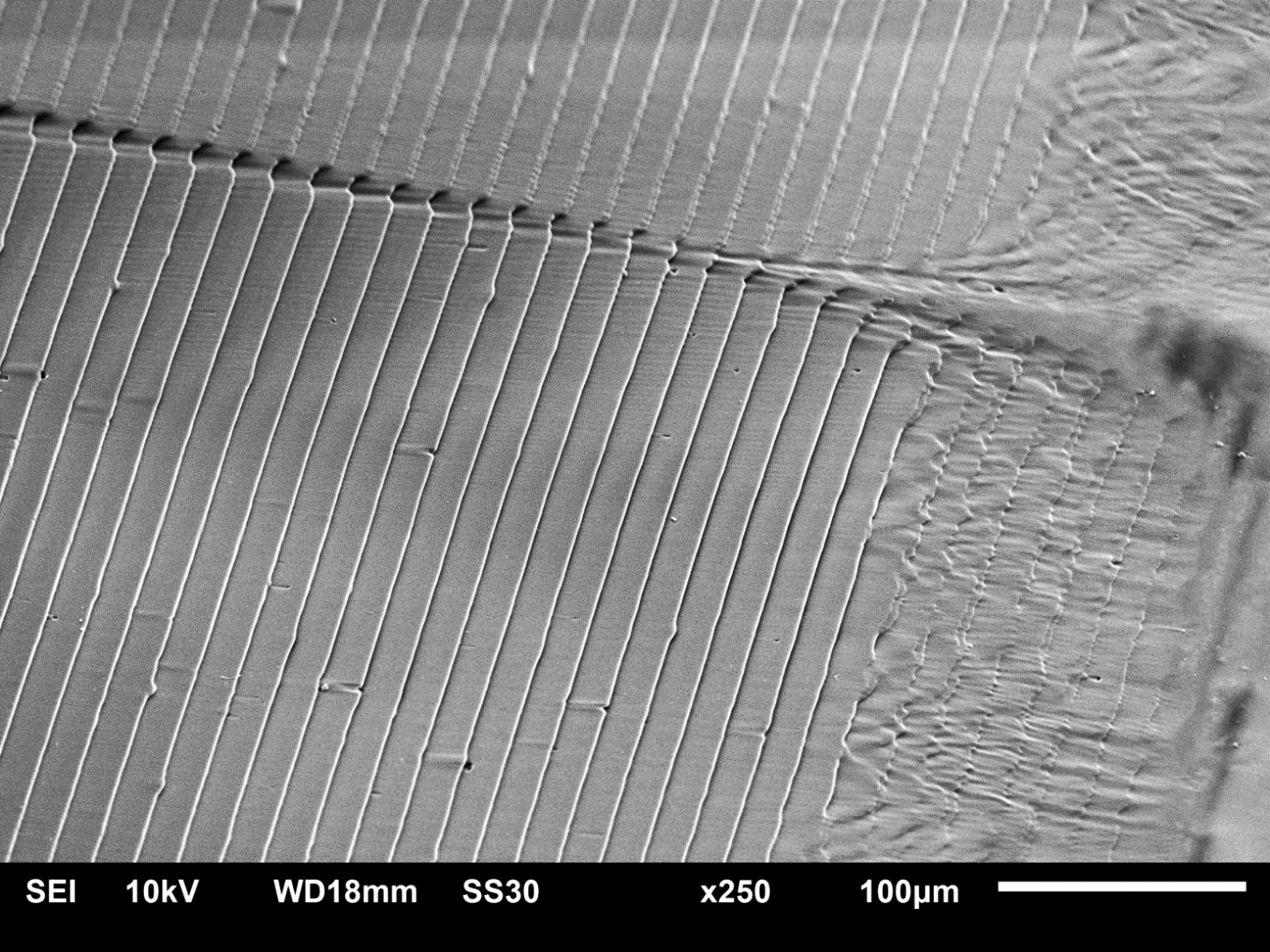


**Figure S1:** Particular of the irradiated area of two adjacent DLIP-pixels, showing irregularities at the pixel-border. The structuring was performed with a laser fluence of 1.32 J/cm², 20 pulses per area and an inclination of 75°.
